# Changes in pallidal neural activity following long-term symptom improvement from botulinum toxin treatment in DYT6 dystonia: a case report

**DOI:** 10.1186/s13256-021-03215-4

**Published:** 2022-01-09

**Authors:** Andrea Giorni, Terry Coyne, Peter A. Silburn, George D. Mellick, Pankaj Sah, François Windels

**Affiliations:** 1 Synaptic Plasticity Laboratory, The Queensland Brain Institute, The University of Queensland, Saint Lucia, QLD 4072 Australia; 2 Asia Pacific Center for Neuromodulation, St Andrews War Memorial Hospital, Brisbane, QLD Australia; 3 Griffith Institute of Drug Discovery (GRIDD), Griffith University, Brisbane, Australia; 4 Brain Research Centre and Department of Biology, Southern University of Science and Technology, Nanshan District, Shenzhen, Guangdong Province People’s Republic of China

**Keywords:** Dystonia, Deep brain stimulation, Globus pallidus, Botulinum toxin, Case report

## Abstract

**Background:**

The globus pallidus internus is the main target for the treatment of dystonia by deep brain stimulation. Unfortunately, for some genetic etiologies, the therapeutic outcome of dystonia is less predictable. In particular, therapeutic outcomes for deep brain stimulation in craniocervical and orolaryngeal dystonia in DYT6-positive patients are poor. Little is known about the neurophysiology of the globus pallidus internus in DYT6-positive dystonia, and how symptomatic treatment affects the neural activity of this region.

**Case presentation:**

We present here the case of a 55-year-old Caucasian female DYT6-dystonic patient with blepharospasm, spasmodic dysphonia, and oromandibular dystonia where single-unit and local field potential activity was recorded from the globus pallidus internus during two deep brain stimulation revision surgeries 4 years apart with no symptomatic improvement. Botulinum toxin injections consistently improved dysphonia, while some of the other symptoms were only inconsistently or marginally improved. Neural activity in the globus pallidus internus during both revision surgeries were compared with previously published results from an idiopathic dystonic cohort. Single-cell firing characteristics and local field potential from the first revision surgery showed no differences with our control group. However, during the second revision surgery, the mean firing rate of single units and local field potential power in the gamma range were lower than those present during the first revision surgery or the control group.

**Conclusions:**

Symptoms related to facial movements were greatly improved by botulinum toxin treatment between revision surgeries, which coincided with lower discharge rate and changes in gamma local field oscillations.

## Background

Dystonia is a hyperkinetic disorder characterized by sustained or intermittent muscle contractions causing abnormal, often repetitive movements and postures. As a movement disorder, dystonias are relatively common, and as with many neurological disorders, both idiopathic and genetically linked forms have been identified [1]. Genetically linked dystonias are generally labelled with the “DYT” nomenclature [2], with the mutations spanning a large spectrum of proteins, including chaperones (DYT1), transcription factors (DYT6), structural proteins (DYT11), and enzymes involved in neurotransmitter biosynthesis (DYT5). Although there is a broad consensus that the basal ganglia are involved in the pathophysiology of dystonia, other brain regions, such as the thalamus and the cerebellum, are considered critical nodes of the circuit underpinning dystonic symptoms [3, 4]. No clear pathophysiology has been identified, but a decrease in γ-aminobutyric acid (GABA)ergic transmission, potentially mimicking the loss of inhibition involved in nongenetic dystonia, has been described in DYT6 mutation bearers [5]. Moreover, it seems likely that certain brain regions, such as the putamen and presupplementary motor area, are involved in dystonia linked to specific genetic mutations [6].

The potential overlap in the pathophysiology of dystonia with or without the DYT6 mutation is unknown. However, these differences could be responsible for the broad variation of therapeutic outcomes observed with globus pallidus internus (GPi) deep brain stimulation (DBS) for DYT6 mutation bearers [7, 8]. Overall, DYT6 patients appear to have less predictable and later onset improvement from DBS than patients with DYT1 or non-DYT dystonia [9]. This outcome seems to be symptom specific, with less improvement in the craniocervical and orolaryngeal region [10].

Here we present data from one patient with DYT6 genetic dystonia, treated with DBS in the posterior GPi. The absence of therapeutic response to the stimulation led to the repositioning of the DBS electrodes twice over a period of 4 years, and microelectrode recordings were obtained during both revision surgeries. Single-cell activity and local field potential (LFP) power from these recordings are compared with results obtained in a GPi-DBS responsive dystonic cohort [11].

## Case presentation

We report the case of a Caucasian female patient with cervical, multisegmental genetic dystonia bearing a c.530T>C *DYT6* variant that leads to the substitution of a leucine to proline residue at codon 177 of the THAP1 protein. See detailed genetic analysis by Newman *et al*. [12]. Dystonia in the hands started at the age of 5 years, followed by oromandibular dystonia at age 11 and later by blepharospasm and spasmodic dysphonia. Informed consent was obtained from the participant included in this study (ethics numbers 2011001012 and 201618196).

The patient first underwent surgery for DBS in 2002 at age 36 years with bilateral electrodes placed in the posteroventral GPi. No microelectrode recordings were obtained during this surgery. After multiple attempts at program optimization over a 12-month period, no benefit was observed, leading the patient to request the stimulation be discontinued.

**Fig. 1:**
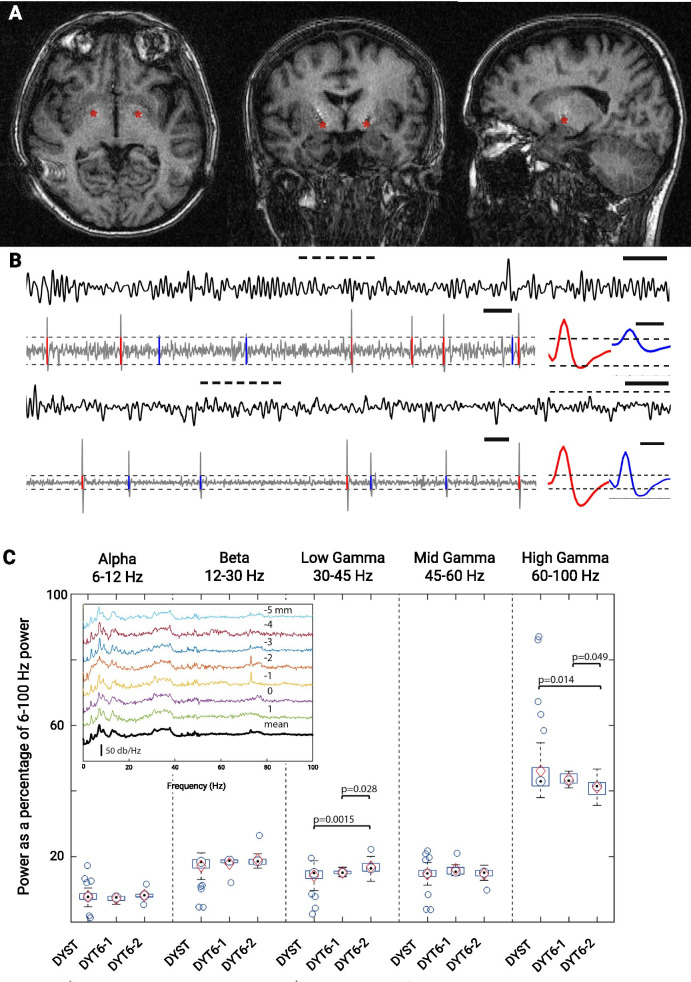
**a** 1.5 Tesla magnetic resonance imaging scans in the axial (left), coronal (middle), and sagittal (left) plan. The red asterisks indicate the lead trajectory in the axial plane and the most distal point reached by the lead in the coronal and sagittal plan. **b** Representative intraoperative microelectrode recordings of LFP (black trace) and units (gray trace) obtained during the DYT6-1 (upper panel) and DYT6-2 (lower panel) surgeries. The epoch of LFP recording marked by the bold dotted line represents the corresponding epoch of unit recording. Gray dotted line around the unit trace represents the detection threshold used to isolate waveforms marked in red and blue. Averaged waveforms for those units are presented on the right side of the trace. Scale bars: LFP trace, 100 ms; single-unit trace, 10 ms; single-unit waveforms, 0.4 ms. **c** LFP band relative power as a fraction of total power within 6–100 Hz band; see text for details of *x*-axis labels. Target symbols represent median, diamonds the mean, and boxes second and third quartiles; whiskers are ± 2.7 standard deviation, and circles are outliers. Spectra of recordings (inset) along an insertion passage in DYT6-2 patient on left side. Recordings were taken at 1 mm step from 5 mm above (top trace) to 1 mm below the planned GPi ventral border; the bottom bold trace is the average

The patient continued to be seen and treated with botulinum toxin (BonT-A) injection of 60 units every 3 months when she was transferred to our care in 2005. This BonT-A treatment optimization led to consistent improvement in spasmodic dysphonia but did not give satisfying and consistent results for facial spasms, oromandibular dystonia, or blepharospasm, and the patient reported only intermittent and limited therapeutic benefit and consequent issues with well-being. As a result, in 2008, the patient was offered revision surgery in an attempt to optimize electrode placement in posteroventral GPi. Microelectrode electrophysiological recordings (MER) obtained during this first revision surgery are identified as our first dataset (DYT6-1, Fig. 1b). DBS program optimization was conducted over the following 9 months in an attempt to improve the patient’s condition but with only marginal success. In parallel, BonT-A injections were extended to bilateral sternocleidomastoids in upper third muscle one site, each side, and splenius capitis bilateral mid portion muscles one site, each side; anterior portion trapezius right side two sites; orbicularis oris bilateral superolateral portion one site; occasional right triceps two sites, 7.5 units each site; each site 10 units BoNT-A, except orbicularis oris 2.5 units each site, every 3 months over the next 4 years.

Over the following years, the patient reported significant symptomatic improvement and improved well-being. The constraints imposed by the BonT-A treatment and some remaining well-being issues, mainly related to facial spasms limiting social interactions, led to the consideration of a second revision surgery. This was performed 4 years later in an attempt to improve the DBS outcome (Fig. 1a) using a more anterior target compared with other dystonic cases [11, 13], and recordings obtained at that time are identified as our second dataset (DYT6-2, Fig. 1b). Despite extensive programming optimization over the following 3 years, there was no apparent benefit from DBS. In that period, BonT-A treatment provided symptomatic relief comparable to what was observed before that second revision surgery.

Microelectrode recordings and analysis have been previously described in detail by Giorni *et al*. (2017) and Tattersall *et al*. (2014) [11, 14]. Revision surgeries were performed after the effects of the BonT-A treatment had worn off to allow for the identification of potential benefit or side effects from DBS during surgery. Magnetic resonance imaging and stereotactic computed tomography scans were co-registered to target the lower border of the posterolateral GPi. The area targeted was 2 mm anterior to the midpoint, 18–21 mm lateral, and 3.5 mm below the anterior commissure posterior commissure (AC/PC) line; lead placement was confirmed by postoperative imaging (P.A.S). MER started only when the patient was awake and responsive to the neurologist’s queries and interventions. The same MER recording protocol was followed for both revision surgeries. MER explorations were made from 5 mm above to 1 mm below the target, in 1 mm steps. Every MER recording was inspected visually, and periods of high-amplitude noise were rejected. Only units recorded in the GPi were considered for our analysis; clustering criteria were identical to those presented by Giorni *et al*. (2017) [11].

The mean spike duration of every unit was calculated as the mean waveform duration of the depolarization deflection at its half-maximum amplitude value. Five methods were used to obtain measures of bursting: Inter-spike interval coefficient of variation (ISIcv), proportion of spikes in in bursts, Burst Index (BI), Pause Index, and L-statistic. For all these methods, a more bursty cell will attain larger values [see Giorni *et al*. (2017) for details]. Power spectral density estimations were made using the Welch method with frequency resolution of 1 Hz. LFP power values for each subband are expressed as a percentage of the total power over the 6–100 Hz band (see Fig. 1C for details). A two-tailed rank-sum test was used to calculate *p*-values.

Single-unit and LFP data recorded from GPi during two revision surgeries (DYT6-1 and DYT6-2) were analyzed and compared with those from the GPi in a cohort of dystonic patients described previously by Giorni *et al*. (2017) (below referred to as dystonic cohort, see Table 1 for details [11]). Notably, this cohort of patients had good therapeutic outcomes with DBS. The recording conditions and analysis methods were identical between the Giorni *et al*. (2017) study [9] and what is presented here.

**Table 1:** Summary statistics of firing characteristics for DYT6 patient at first surgery (DYT6-1), second surgery (DYT6-2), and primary dystonic cohort

Mean, median, standard deviation	DYT6-1 (*n* = 7)	DYT6-2 (*n* = 9)	Dystonic cohort (*n* = 32)
Spike width (μs)	194.46, 199.25, 294.16	192.30, 185.35, 320.95	199.48, 189.64, 418.38
Mean firing rate (Hz)	54.28, 34.84,58.00	12.43^*$^, 9.19, 8.59	47.48, 33.91, 46.90
Median firing rate (Hz)	68.90, 47.11, 58.79	35.47, 13.35, 55.59	70.30, 50.37, 59.48
ISI CV	0.99, 0.96, 0.20	0.87, 0.83, 0.26	1.23, 1.11, 0.68
Proportion of spike in bursts	0.15, 0.18, 0.13	0.30, 0.28, 0.25	0.35, 0.32, 0.25
L-statistics	5.71, 6.00, 1.11	5.77, 6.00, 1.85	6.31, 6.00, 2.02
Burst Index	3.10, 3.26, 1.12	6.51, 2.27, 9.95	3.10, 2.75, 1.76
Pause Index	0.12, 0.11, 0.04	0.17, 0.10, 0.28	0.11, 0.10, 0.07

Mean, median, and standard deviation are reported in this order

Statistical significance is indicated with * for difference between first and second surgery and $ for comparison between the second surgery and dystonic cohort

The median half-widths of action potentials recorded during DYT6-1 and DYT6-2 surgery were not significantly different from each other or from units recorded in the dystonic cohort at 199.3 µs (DYT6-1), 185.4 µs (DYT6-2), and 189.6 μs (dystonic cohort). The mean discharge frequency of units recorded during DYT6-1 was 54.3 ± 58.0 Hz (*n* = 7), similar to that seen from our dystonic cohort (47.5 ± 46.9 Hz; *n* = 32); however, it was significantly lower for units recorded during DYT6-2 (12.4 ± 8.6 Hz; *n* = 9) (*p* = 0.0012 compared with DYT6-1 and *p* = 0.0029 compared with the dystonic cohort). Notably, the range of firing rates recorded at DYT6-1 (19.8–184.9 Hz) and DYT6-2 (6.4–34.7 Hz) in this DYT6 patient overlapped completely with the range observed in the dystonic cohort (3.9–195.8 Hz). Several metrics were used to evaluate bursting activity from the spike trains in our three datasets (see “Methods”), though no significant difference was found between them (Table 1).

We analyzed LFPs recorded in GPi and compared the mean power in the alpha (6–12 Hz), beta (12–30 Hz), and low-, mid-, and high-gamma (30–45 Hz, 45–60 Hz, 60–100 Hz) bands. Low-gamma band power at DYT-1 was 15.4 ± 0.8 (0.29), similar to that in our separate dystonic cohort 14.4 ± 3 (0.39) but was significantly higher (*p* = 0.028 compared with DYT6-1 and *p* = 0.002 compared with the separate dystonic group) in DYT6-2 (17.1 ± 2.5 (0.72). High-gamma power was lower for the second surgery (41.7 ± 3 (0.9)) when compared with the first surgery (44.2 ± 1.8 (0.65); *p* = 0.0491) and the other primary dystonic cohort (46.9 ± 9.4 (1.2); *p* = 0.0106).

## Discussion

Initially developed as a treatment for Parkinson’s disease, DBS is now also used for other movement disorders [15] and some psychiatric conditions [16]. This case report presents data from patients with genomic DYT6 dystonia and reveals changes in GPi neurophysiology that coincide with symptomatic improvement, though not as a result of DBS. Spike train characteristics and LFP power recorded during the first revision surgery were not significantly different from values obtained from our previously published cohort of dystonic patients [11], and were also comparable to those reported from other dystonic cohorts of genetic or nongenetic origin [7, 17] treated by DBS and BonT injection for certain cases. Thus, while the therapeutic outcome of DBS was very different, it appears that the discharge properties of GPi neurons in DYT6 is not different from those in other dystonic groups. However, coinciding with symptomatic improvement following BonT-A injections, there were clear differences in the discharge rate of single units and in LFP gamma power in the GPi of this DYT6 patient. Overall discharge frequency was lower, and there were changes in both high- and low-gamma LFP power between the first and second revision surgeries. Interestingly, there were no changes in the 3–10 Hz range, consistent with the lack of therapeutic response to the DBS [18, 19]. Neurophysiological differences between DYT6-2, DYT6-1, and the other primary dystonic cohort are limited to firing rate and gamma power, which leads us to believe that these differences are not due to the preimplantation of the DBS lead. The divergence of neuronal firing rates suggests that an external factor may be influencing the activity of some cells. We speculate that feedback from muscle could be linked to this decreased activity. A study combining GPi LFP oscillations and sternocleidomastoid electromyogram coupling in dystonic patients has shown that GPi activity receives a net drive from the pallidum but also clearly established that feedback from the muscle also has an impact [20]. Moreover, voluntary movement has been shown to specifically affect gamma oscillations in GPi of dystonic patients [21]. Over time, the functional recovery obtained as a result of by BonT-A injections could influence plastic changes in GPi through changes in muscle feedback during voluntary movement.

Thus, we speculate that the neurophysiological changes reported here may originate from the BonT-A treatment received by the patient between the two surgeries, a phenomenon already reported for the peripheral nervous system [22]. The effective treatment of dystonic symptoms has a correlate at the central nervous system level that is not limited to cortical and subcortical representations of the treated muscles [23]. BonT-A treatment has also been shown to induce a reorganization of cortical and subcortical networks involving GPi [24]. There does not seem to be a direct link between the genotype of the patient [12] and the current findings. The same changes were observed at revision surgery for non-DYT6 dystonic patients where DBS was combined with botulinum injection [25].

## Conclusion

Although the results presented here come from a single case, functional plasticity could be responsible for the differences in pallidum activity observed before and after dystonic symptoms were improved by BoNT-A treatment.

## Data Availability

Consent was obtained from the patient solely for the purpose of this study and provided to the authors under the ethics applications cited above. As such, the data can only be made available upon re-consenting the patient.

## Abbreviations

AC/PC: Anterior commissure/posterior commissure
BI: Burst Index
BoNT-A: Botulinum toxin
DBS: Deep brain stimulation
ISIcv: Inter-spike interval coefficient of variation
GPi: Globus pallidus internus
LFP: Local field potential
MER: Microelectrode recording
